# From Synaptic Physiology to Synaptic Pathology: The Enigma of α-Synuclein

**DOI:** 10.3390/ijms25020986

**Published:** 2024-01-12

**Authors:** Kaja Nordengen, Cecilie Morland

**Affiliations:** 1 Department of Neurology, Oslo University Hospital, 0424 Oslo, Norway; 2 Section for Pharmacology and Pharmaceutical Biosciences, Department of Pharmacy, The Faculty of Mathematics and Natural Sciences, University of Oslo, 1068 Oslo, Norway

**Keywords:** synuclein, synapse, GABA, glutamate, acetylcholine, serotonin, dopamine, norepinephrine

## Abstract

Alpha-synuclein (α-syn) has gained significant attention due to its involvement in neurodegenerative diseases, particularly Parkinson’s disease. However, its normal function in the human brain is equally fascinating. The α-syn protein is highly dynamic and can adapt to various conformational stages, which differ in their interaction with synaptic elements, their propensity to drive pathological aggregation, and their toxicity. This review will delve into the multifaceted role of α-syn in different types of synapses, shedding light on contributions to neurotransmission and overall brain function. We describe the physiological role of α-syn at central synapses, including the bidirectional interaction between α-syn and neurotransmitter systems.

## 1. Introduction

Alpha-synuclein (α-syn) is abundant in a healthy human brain, where it is concentrated in presynaptic nerve terminals. The α-syn protein is found in most brain regions and in different synapse types, leaving a puzzling question in its wake: which function does this enigmatic protein have at the synapse? The high prevalence and ubiquitous expression of α-syn implies that it plays important roles in a healthy brain. Synaptic α-syn is not merely a bystander but is instead suggested to orchestrate a symphony of synaptic processes that are important for neural health. Nevertheless, it is not the loss of normal function that has attracted the most attention in the search to understand diseases involving α-syn. Rather, it is the devastating consequences of its misfolding. Research focus has been on the toxicity of the pathological α-syn species. The misfolding of α-syn and its accumulation in Lewy bodes and/or Lewy neurites lie at the heart of neurodegenerative diseases like Parkinson’s disease (PD) and Lewy body dementia (DLB). Glial cytoplasmic inclusions of α-syn are associated with multiple system atrophy (MSA) [1,2]. In PD, α-syn accumulation seems to follow a sequential order, starting in the lower brain stem and propagating through the midbrain to the neocortex; this forms the basics of the six stages described by Braak [3]. In the early Braak stages, α-syn accumulation and neurodegeneration co-occur in, e.g., the raphe nuclei in the brain stem [4,5], where neurodegeneration of serotonergic neurons can lead to depressive symptoms [6]. Other non-motor symptoms in PD, like REM sleep behavior disorder, are linked to the degeneration of cholinergic neurons in the pedunculopontine nuclei (PPN) in pons [7]. The classic motor symptoms obligatory for the PD diagnosis, such as rigidity, bradykinesia, and tremor [8], however, do not present themselves until α-syn accumulation reaches the midbrain and degeneration of dopaminergic neurons occurs in the *substantia nigra*. Although these cardinal symptoms of PD are ascribed primarily to the nigrostriatal deficiency of dopamine, PD symptoms like postural instability and the characteristic shuffling gait may partly result from a central deficiency in norepinephrine signaling from the *locus coeruleus* [9,10]. Also, orthostatic hypotension, which affects about 40% of PD patients, likely reflects an impairment of the autonomic responses due to reduced norepinephrine signaling in the sympathetic ganglia. In DLB, on the other hand, α-syn accumulation affects cortical areas earlier in the disease trajectory than it does in the case of PD, reflected by the diagnostic criteria that emphasize cognitive symptoms over motor symptoms [11]. While α-syn accumulation is mostly neuronal in both PD and DLB, one of the neuropathological characteristics of MSA is additional α-syn accumulation in oligodendrocytes [12,13]. Misfolding and accumulation of α-syn in Lewy bodies, Lewy neurites, and glial cytoplasmic inclusions are critical hallmarks of synucleinopathies; nevertheless, only a few genes associated with these diseases are known to directly affect α-syn [14,15,16]. Hence, the majority of PD, DLB, and MSA cases occur in the presence of normal/unmutated α-syn. What triggers α-syn aggregation in the human brain is still a mystery, and so are many of the mechanisms which result in Lewy body formation and the death of specific neuronal populations in this disease.

The diagnostic landscape is further complicated by the paradox of α-syn’s abundance. Unlike with Alzheimer’s disease, where the protein levels (of amyloid β) in the cerebrospinal fluid (CSF) can be measured and used directly for diagnosis, conventional CSF α-syn ELISA has proven unsuitable as a diagnostic biomarker. This may be due to the abundant presence of α-syn also in the healthy human brain. In synucleinopathies, measuring α-syn’s propensity for misfolding and aggregation has necessitated innovative approaches, such as real-time quaking-induced conversion (RT-QuIC) [17,18].

The focus of the present review is on the function of α-syn within different synapses; however, the prion-like spreading of α-syn from synapse to synapse is worth mentioning (for a comprehensive review of this matter, see [19]). Misfolded monomeric α-syn as well as aggregated α-syn can be secreted from damaged neurons through exosomes/extracellular vesicles [20] or in the free form. The presence of α-syn in exosomes in the CSF, or even in the blood, is increased in PD patients and has been suggested as a biomarker for PD [21]. Released α-syn species can be taken up by neighboring healthy synapses, where the misfolded α-syn may seed Lewy body formation [22]. Direct transfer of misfolded α-syn through tunneling nanotubes has been suggested [23], but in vivo evidence is lacking.

The presence of extracellular α-syn may in itself lead to a spectrum of effects, including calcium dysregulation and synaptic dysfunction, and is further hypothesized to contribute to neurodegeneration [24,25] and cognitive impairments [26,27,28]. Secreted α-syn is shown to induce calcium influx though its interaction with and disruption of membranes [25]. Both monomeric [24] and oligomeric [25] α-syn disrupt calcium signaling, but only the oligomeric form has been reported to induce neurodegeneration [24]. Decreased expression of calcium buffer proteins is directly correlated with cognitive impairment in model animals [26]. Extracellularly introduced α-syn may modify both pre- and postsynaptic activity through the disruption of lipid rafts [29,30].

There is an ongoing discussion regarding the reason behind the synaptic degeneration in PD, where the direct toxic effects of aggregating α-syn and α-syn’s loss of function at the synapse due to aggregation are among the hypotheses. Also, differences in vulnerability between neurons seem to be of relevance, a vulnerability that goes across transmitter systems; the early-affected neurons in PD are the norepinephrinergic neurons in the locus coeruleus, serotonergic neurons in the raphe nuclei, and cholinergic neurons in the basal forebrain, in addition to the most widely known dopaminergic degeneration of the substantia nigra pars compacta [31]. Common features between these neurons are prominent calcium currents, low intrinsic calcium buffering capacity, sustained spontaneous spiking, and broad spikes, which together possibly cause their vulnerability as pointed out by Surmeier and colleagues [31]. Although synaptic degeneration and α-syn aggregation in PD have been described in synapses using each of the six neurotransmitters covered in this review, there are also more resilient synapses using the same neurotransmitters [31].

In this review, we will explore the physiological role of α-syn in normal neurotransmission across transmitter systems, as well as how pathological α-syn variants and their aggregates interfere with neurotransmission in PD. Although numerous review articles have explored the general function of α-syn in synapses, the extensive heterogeneity among synapses in the brain necessitates a focused summary of the literature on the function of α-syn in individual synapse types. The current review delineates significant variations across different synapse types and even within synapses sharing the same neurotransmitter, underscoring the remarkable diversity in the role of α-syn at various synaptic sites. Investigating the normal role of α-syn offers insights into fundamental processes of neuronal communication, protein function, and synapse biology, which have broader implications beyond specific diseases. Despite that, a comprehensive understanding of α-syn function in different synapses not only underpins healthy neurotransmission, but also holds promise in detecting subtle synaptic alterations occurring in synucleinopathies prior to the onset of overt Parkinsonian symptoms.

## 2. Aggregation and Toxicity of α-Synuclein

The 140-amino acid protein α-syn is encoded by the *SNCA* gene. Structurally, α-syn consists of three main regions: (1) The N-terminal region (residues 1–60), which may form an α-helix which is essential for binding to curved phospholipid bilayers, including the membrane of several organelles. (2) The non-amyloid component (NAC) region (residues 61–95), consisting of a central hydrophobic segment flanked by polar and charged residues. This region is known to play a critical role in protein-to-protein binding and hence the aggregation and toxicity of α-syn. (3) The acidic C-terminal region (residues 96–140), which has been found to possess chaperone-like activity. The protein is ascribed to the class of intrinsically disordered proteins (the disordered region is at residues ~101–140), meaning that it does not have a stable 3D structure. Instead, α-syn exists in an equilibrium between a soluble cytosolic form, which is highly unstructured, an aliphatic α-helix-rich structure bound to phospholipid membranes, and a β-sheet-rich structure prone to oligomerize, form fibrils, and deposit in Lewy bodies or Lewy neurites [32].

The monomeric α-syn protein is highly dynamic and can adapt to various conformational stages, each exposing slightly different amino acid sequences at the surface. These amino acid sequences may bind to complementary amino acid sequences on other α-syn monomers. Hence, the dynamic confirmation results in a myriad of α-syn monomers with different propensities to aggregate into dimers, trimers, and further into oligomers. One belief is that all of these monomeric conformations are in equilibrium, and their life span depends on their stability and ability to involve in larger aggregates. The matter is further complicated by post-translational modifications of α-syn, which may result in monomers that are more or less prone to be involved in aggregates and/or toxicity. Examples of post-translational modifications of α-syn are acetylation, nitration, oxidation, and ubiquitination. Whether or not these post-translationally modified α-syn proteins contribute to driving disease progression in synucleinopathies is not known, but several types have been reported in Lewy bodies found in the brains of PD patients. In general, the post-translational modifications presented above introduce functional groups that make the monomers less compatible with fibril formation. The ability of α-syn monomers to form multimers may in fact be essential for the toxicity of α-syn. For instance, α-syn mutants that were incapable of multimerization have been reported to accumulate in the cells as monomers, resulting in enhanced clustering of vesicles of different sizes and the formation of cytoplasmic inclusions [33]. Furthermore, α-syn monomers where the effects of the PD-linked E46K was enhanced induced vesicle clustering even more. Adding hydrophobicity to the hydrophobic half of the α-helix of non-multimerization α-syn, interestingly, restored multimerization, reversed the strong membrane binding, and prevented vesicle clustering. These findings, hence, pinpoint α-syn monomers as the major source of toxicity, not primarily through the formation of oligomers and fibrils.

**Figure 1:**
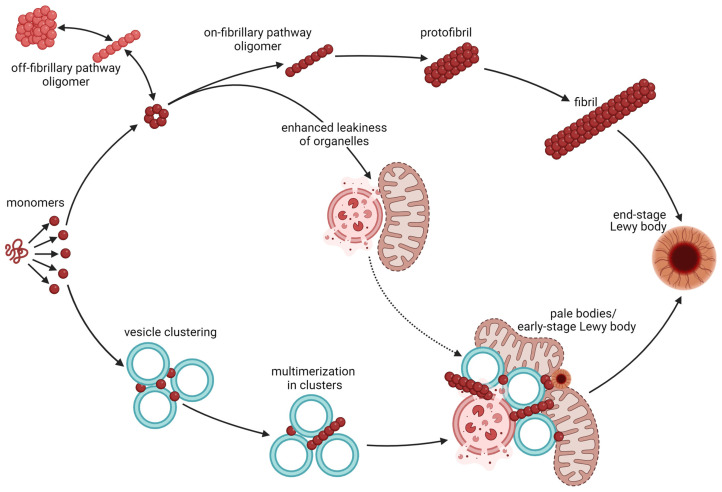
Aggregation of α-syn and the formation of Lewy bodies. Monomers of α-syn are intrinsically disordered and hence exist in an equilibrium of 3D structures with different propensities to aggregate, bind lipids, and bind other proteins. The classical view is that monomers form (black arrows) on-pathway oligomers (dark red) as well as off-pathway oligomers (light red). These α-syn species are in equilibrium, but only the on-pathway oligomers readily form protofibrils and fibrils that further contribute to the formation of Lewy bodies. Classical Lewy bodies consist of a dense core of granular proteins, surrounded by a clearer halo consisting of radially oriented filaments and with pigment granules at the surface (end-stage Lewy body). Newer observations, however, suggest that monomeric α-syn with a high propensity to bind curved lipid membranes induces clustering of synaptic vesicle (vesicle clustering) without prior aggregation of α-syn into oligomers or fibrils. Binding of α-syn to the curved vesicle membranes may in turn propel the aggregation (multimerization in clusters) of α-syn and the assembly of disrupted organelles and membrane fragments into pale bodies (pale bodies/early-stage Lewy body). Pale bodies often contain fragmented mitochondria, lysosomes, and other organelles along with fibrillary α-syn. Most of them also have small classical core-and-halo Lewy bodies along the edge. The pale bodies are believed to progress into classical Lewy bodies (end-point Lewy bodies) as the disease progresses and may hence be referred to as early-stage Lewy bodies. Oligomers of α-syn may induce permeabilization of membranes of mitochondrial and other organelles, hence enhancing leakiness of these. This does not only enhance toxicity, but may (dotted arrow) increase the accumulation of such disrupted organelles in pale bodies. This figure was created by the authors, using biorender.com.

Oligomers of α-syn also exist in a large variety of forms with different morphological, structural, and size features [34,35]. The oligomers themselves do not precipitate in cells under physiological conditions, and hence are often referred to as “soluble” to distinguish them from the insoluble fibrils they may contribute to forming (Figure 1). The literature is not consistent with respect to whether synuclein oligomers or fibrils are more neurotoxic. In a seminal paper by Winner and colleagues [36], overexpression of mutant α-syn that induced the formation of large oligomers was shown to cause the loss of dopaminergic neurons of *substantia nigra* much more efficiently than mutations, resulting in the rapid formation of α-syn fibrils on a by-weight basis. On a by-particle basis, however, α-syn aggregates have been found to be more toxic than soluble oligomers [37,38].

Furthermore, soluble α-syn oligomers may induce membrane permeabilization, leading to leaky plasma membranes, mitochondrial membranes, and membranes of the endoplasmic reticulum. Disruption of the mitochondrial membrane, with a concomitant loss of the proton gradient needed to drive oxidative phosphorylation, is part of the well-known toxicity triangle, which also involves an enhanced formation of reactive oxygen species (ROS), and an increased release of calcium to the cytosol. All of these features have been reported to be enhanced in response to α-syn oligomers [39,40,41,42,43].

In synapses, soluble α-syn oligomers have been reported to induce the permeabilization of synaptic vesicles as well as decreasing synaptic firing and decreasing neuronal excitability. Synaptotoxicity may result from this and may even drive neuronal death. Contributing to a vicious cycle of proteinopathies, α-syn oligomers may inhibit neuronal protein degradation through autophagy and the ubiquitin–proteasomal system [44]. Furthermore, oxidized and misfolded α-syn may itself undergo vesicular release via exocytosis, in particular during cellular stress [45]. Extracellular α-syn has been shown to inhibit microglial autophagy via activation of the Toll-like receptor 4 (Tlr4)-p38-Akt-mTOR signaling pathway [46]. Overexpression of human α-syn in a mouse model where microglial autophagy was turned off resulted in enhanced neuroinflammation, loss of dopaminergic neurons of the midbrain, and impaired motor function [47]. Whether these effects are mediated predominantly by α-syn monomers or soluble oligomers remains unknown. Distinguishing the toxic effects of the three α-syn species is challenging unless it is addressed specifically, as most oligomers form protofibrils and most protofibrils progress into fibrils. Some oligomers are, however, likely off the aggregation pathway and hence do not contribute to synucleinopathies (Figure 1).

In two seminal papers, Lewy bodies of the *substantia nigra* were described as inclusions deep within the cytoplasm, consisting of a dense core of predominantly granular proteins, surrounded by a clearer halo with radially oriented filaments and pigment granules at the surface [48,49]. A small number of Lewy bodies closer to the cell surface were, however, found to be larger in size and having a more uniform structure containing irregularly placed fibrillary material, and lacking the core and the halo [48]. Due to their morphological appearance, the latter occlusions are often referred to as pale bodies. Later, α-syn was found to be the major filamentous component of both Lewy bodies and pale bodies [1], and probably plays a central role in their assembly. Furthermore, the morphology and molecular content of Lewy bodies vary between different brain regions [50]. The current belief in the field is that the heterogeneity of Lewy pathology may represent disease progression, with the classical core-and-halo type of Lewy bodies representing the final stage [50]: The first sign of synuclein pathology is a diffuse cytoplasmic immunoreactivity for α-syn in morphologically healthy-looking neurons. Later, irregularly shaped structures that present uneven staining for α-syn can be detected [50]. In the substantia nigra, these are often present in poorly pigmented neurons. At the third stage, pale bodies are visible. Condensations or small Lewy bodies are often found along the periphery of the pale bodies, and these gradually develop into typical Lewy bodies while the pale bodies disappear [50]. The final stage is the typical Lewy bodies characterized by a distinct core and a surrounding halo. Supporting this belief, pale bodies outnumber classical Lewy bodies at earlier stages of the disease, and only a few of the inclusions found in PD brains post mortem are of the traditional core-and-halo type [50].

Despite the fact that α-syn is involved in the assembly of Lewy bodies, these occlusions contain a myriad of proteins and other molecules in addition to α-syn [50]. Recent progression in the field has led to the understanding that most Lewy bodies consist of fragmented membranes, organelles and vesicles with a varying amount of protein filaments [51]. The findings of Dettmer and colleagues [33] suggest that α-syn monomers induce the clustering of vesicles in the synapses and the formation of cytoplasmic inclusions [33], which further supports this notion. Hence, it appears that the propensity of α-syn to bind curved lipid membranes may propel the assembly of disrupted organelles and membrane fragments into Lewy bodies. Dysfunctional organelles or impaired organelle trafficking may, however, also drive the assembly (Figure 1). Interestingly, the binding of monomeric α-syn to membranes within forming Lewy bodies has been found to favor α-syn aggregation [33].

In (Thy-1)-h[A30P] α-syn transgenic mouse models of PD, antibodies targeting soluble α-syn oligomers have been reported to protect nigral neurons against toxicity [52]; but whether this is a direct effect of reduced oligomer-induced toxicity or occurs secondarily via reduced protofibril and/or fibril formation and aggregation in not resolved.

Recently, the effectiveness of immunotherapy with monoclonal antibodies targeting aggregated forms of α-syn have failed to improve clinical outcomes in randomized control trials [53,54]. It should be noted that these studies do not exclude α-syn immunotherapy as a suitable strategy, as the studies generally are based on few patients (n = ca 100 per treatment group), have a short follow-up time (less than two years), and have end-point measures at a lower rate of precision than comparable studies for anti-amyloid therapy [55,56].

## 3. Synaptic Location and Function of α-Synuclein

In the brain, α-syn localizes specifically to presynaptic sites [57,58,59,60]. The interaction between α-syn and lipid rafts is important for the synaptic localization of α-syn [61,62], and disruption of this association redistributes α-syn from the synaptse [61]. While the majority of α-syn resides intracellularly, a robust body of research also supports the presence of extracellular forms of α-syn [20,63,64,65,66].

Immunohistochemical analysis has revealed that α-syn appears very early in brain development. The protein is formed in neuroectodermal cells of the telencephalon at embryonic day 12, becoming notably more pronounced by embryonic day 15. This is even earlier than the synaptic vesicle (SV) protein synaptophysin [67]. Despite this very early expression in brain tissue, α-syn finds it place at the synapse at a later stage. A shift from neuronal cell bodies to the neuropil was noted at postnatal day 7 in an intact mouse brain, indicative of a potential synaptic localization [67]. Thereafter, α-syn maintains a consistent presence in nerve terminals across the mature mammalian brain [61,67]. In cultured neurons from embryonic rat hippocampus, α-syn accumulated in nerve terminals quite late during synaptogenesis and after functional synapses had been formed and other key synaptic proteins had found their synaptic location [60]. Further suggesting that α-syn is not essential for synapse formation, the protein is restricted to vertebrates, while non-vertebrates develop their nervous systems—including synapses—in the absence of α-syn. Additionally, α-syn-deficient mice show normal brain development without detectable deviations in morphology, number of different cell types, or synapse density [68]. The α-syn-deficient mice show, however, decreased numbers of SVs in the reserve pool [68], consistent with reduced neurotransmitter release in response to prolonged, high-frequency stimulation, and an enhanced rate of recovery during paired-pulse stimulation [68,69]. Although not essential for the exocytotic release of neurotransmitters, α-syn affects neurotransmission in several ways, which may differ between different types of synapses.

### 3.1. General Mechanisms of Synaptic α-Synuclein

**Figure 2:**
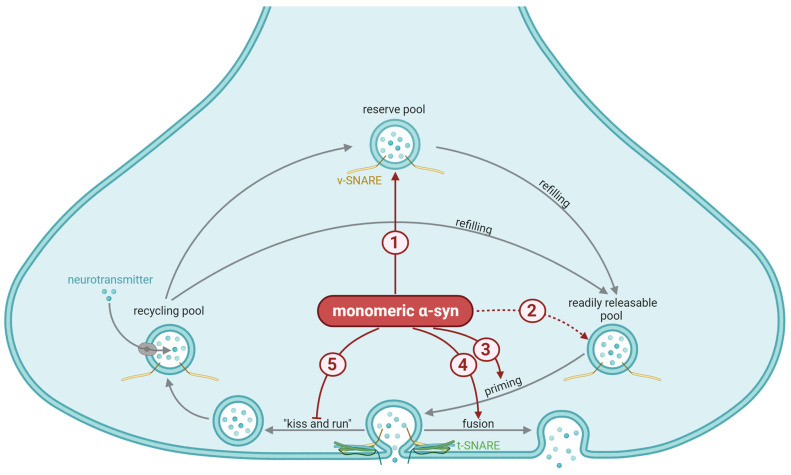
General synaptic mechanisms affected by α-synuclein (α-syn). The vesicles in the nerve terminal are categorized into the readily releasable pool, which undergoes priming (grey arrow) prior to exocytosis; the recycling pool, which is responsible for refilling the readily releasable pool during moderate levels of stimulation (grey arrow); and the reserve pool, which refills the readily releasable pool during more longstanding stimulation (grey arrow). The importance of the reserve pool is controversial, as this pool is small—or even absent—in intact brain tissue. Priming involves a partial assembly of the soluble N-ethylmaleimide-sensitive factor attachment protein receptor (SNARE) complex, meaning that the vesicular SNARE (vSNARE, yellow) and the target membrane SNARE (tSNARE, green) bind together to facilitate exocytosis. Priming and docking of the vesicles are orchestrated by numerous proteins. Exocytosis may occur either via the complete fusion of the vesicular membrane with the plasma membrane (fusion) or a partial fusion that creates a narrow aqueous fusion pore through which neurotransmitters may diffuse before the vesicles quickly disengage from the plasma membrane (“kiss-and-run”). The suggested mechanisms stimulated (red arrows) or inhibited (red blunted arrows) by monomeric α-syn involve: (1) Maintaining the reserve pool of vesicles (a reduced size of this pool has been reported in response to depletion of α-syn). Inconsistent with the previous mechanisms, (2) a selective increase in the number of vesicles in the readily releasable pool in response to overexpression of α-syn has been seen in vitro (dotted arrow indicates uncertainty). Through interaction with several of the priming chaperones, α-syn regulates (3) SNARE complex assembly and exocytotic release of neurotransmitters. The presence of α-syn promotes dilation of the fusion pore, hence (4) shifts exocytosis toward a higher prevalence of full vesicle fusion and (5) a lower probability of kiss-and-run events. This figure was created by the authors, using biorender.com.

The vesicles in the nerve terminal are categorized into three pools based on their function and location. These are the readily releasable pool, the recycling pool, and the reserve pool (Figure 2). The readily releasable pool consists of vesicles that are docked at the cell membrane and are readily available for release upon stimulation. This pool is relatively small and quickly becomes depleted. The recycling pool is situated close to the cell membrane and undergoes cycling during moderate levels of stimulation, ensuring that the rate of vesicle release does not exceed the rate of vesicle formation. The recycling pool is larger than the readily releasable pool, but it takes longer to mobilize these vesicles. The importance of the reserve pool is controversial, as this pool is large in neurons grown in vitro, but is small—or even absent—in mature synapses of intact brain tissue [70,71]. Consistent with the presynaptic location, α-syn plays a pivotal role in the regulation and maintenance of SV recycling and neurotransmitter release. A multitude of in vitro and in vivo experiments exploring α-syn loss or gain of function have uncovered its influence on various stages of SV trafficking [72,73], encompassing processes such as docking [74,75], priming, fusion of release-competent SVs [76], and the re-clustering of SVs after neurotransmitter release [72,73,77].

Synaptic release of neurotransmitters consists of several steps, and α-syn has been demonstrated to be involved in most of these. First, specific transporter proteins in the vesicular membrane are responsible for the loading of neurotransmitter into the vesicle. Vesicular loading of monoamine transmitters occurs through the vesicular monoamine transporter 2 (VMAT2), the activity of which is inhibited by α-syn [78]. Although this has only been shown to affect vesicular dopamine uptake in dopaminergic neurons, it likely applies to the other monoamines as well. Vesicular transporters for other neurotransmitters, like acetylcholine, glutamate, and GABA has, to the best of our knowledge, not been reported to be affected directly by α-syn. The vesicular GABA transporter (VGAT) protein has, however, been associated with α-syn toxicity [79,80], but without evidence of direct effects in human synapses.

Next, the loaded vesicles dock near the release sites to join the readily releasable pool. They further undergo priming, in which the docked vesicles are prepared to fuse with the plasma membrane in response to calcium influx. In a rodent hippocampus, depletion or partial depletion of α-syn has been reported to cause a reduction in the reserve/resting pool of vesicles while leaving the readily releasable pool unaffected [69,81]. In line with this, prolonged, lower-frequency stimulation that would be expected to deplete the reserve pool was significantly impaired in the α-syn-deficient mice compared to the wild-type mice. In contrast, synaptic responses to brief high-frequency stimuli, which was sufficient to exhaust docked synaptic vesicles but not the reserve pool, were unaffected by the lack of α-syn [69]. Further suggesting a role of α-syn in hippocampal synapses, Lie and colleagues found that α-syn affected transmitter release from the presynaptic terminal during plasticity [82]. In dopaminergic nerve terminals of the striatum, paired-stimulus depression underlies one form of plasticity. Mice deficient in α-syn have been reported to exhibit faster recovery from such paired-stimulus depression, consistent with reduced plasticity [68]. Together, these studies suggest a role for α-syn in the regulation of the reserve or resting pools of vesicles across different synapse types, which affects plasticity mechanisms. Overexpression of α-syn in vitro, however, has been reported to induce an increase in vesicles in the readily releasable pool, without affecting the other pools of vesicles, and in these cells, overexpression of α-syn inhibited vesicle priming [83].

Priming is the intricate process that prepares the vesicle for exocytosis. It involves a partial assembly of the SNARE complex and is orchestrated by numerous proteins in symphony. Among those are the SNARE protein synaptobrevin-2/vesicle-associated membrane protein 2 (VAMP2), synapsins, Munc13 isoforms, Munc18 isoforms, several Rab proteins, Rab-interacting molecule (RIM), and RIM-binding protein. A detailed description of the priming process is beyond the scope of this review, but some key players that interact with α-syn are worth mentioning. Furthermore, alterations in many of these proteins have been observed in brain samples from PD patients and various transgenic mouse models expressing human α-syn [84]. Rab proteins form a family of small GTPases that play a crucial role in vesicle trafficking, priming and membrane fusion events within cells. Binding α-syn to several of these has been reported, including—but not restricted to—Rab3a and Rab8a. Binding α-syn to Rab3a is potentiated in response to interactions with the surface of SVs and results in a decreased GTP hydrolyzing activity [85]. Interestingly, α-syn species carrying PD-linked phosphorylation at ser129 showed greater inhibition of Rab3a GTPase activity than their unphosphorylated equivalents [85]. Rab3A further modulates the function of Munc13-1 and Munc18-1 [86]. Both of these are key chaperones required for the assembly of the SNARE complex. Munc18-1 probably acts downstream of Munc13-1, but both proteins have been reported to interact with syntaxin-1, VAMP2, and SNAP25 to ensure the proper assembly and stability of the SNARE complex and ultimately trigger SV fusion for synaptic transmission. Through interaction with several of these proteins, α-syn regulates SNARE complex assembly and exocytosis [72,74,75,76,87]. Work from the laboratories of Roy and Gitler suggests that the synapsins also act as key regulators of the synaptic effects of α-syn [88,89]. The interaction between α-syn and these release-regulating proteins provides a potential mechanism through which α-syn affects neurotransmitter release in health and pathology. Interestingly, through their binding to α-syn, many of the proteins that regulate the exocytosis of SVs affect α-syn aggregation and/or toxicity. For instance, several studies suggest that Rab proteins are key players in the synaptopathy seen in PD [90,91].

Finally, vesicular release of neurotransmitters involves the fusion of the membrane of the SVs with the plasma membrane. This occurs either through a complete fusion of the two membranes, or through a partial fusion where the neurotransmitters are released through a narrow aqueous fusion pore before the vesicle disengages from the plasma membrane. The latter process is referred to as “kiss-and-run” [92]. SNAREs on the target membrane (t-SNARES) or on the vesicle (v-SNAREs) are responsible for docking the vesicle. These proteins do not, however, appear to control the dilatation of the fusion pore [87] and hence whether the SVs undergo kiss-and-run or fully collapse into the plasma membrane to release their content.

Binding to both the lipid membrane and membrane-associated proteins of synaptic vesicles, α-syn interacts with SVs in multiple ways. The details regarding the binding of α-syn to the phospholipid membrane have, for methodological reasons, been hard to prove. The α-syn protein does not have a transmembrane domain or a lipophilic anchor and hence dissociates from the SVs during isolation steps, etc. Nevertheless, α-syn shows a propensity to bind membranes with high curvatures, such as SVs. Possibly, the close proximity between α-syn and highly curved membranes with acidic phospholipid headgroups pushes the N-terminal region of α-syn into an amphipathic α-helical structure, which burrows into the lipid bilayer [93,94,95].

Not only may α-syn be attracted to curved membranes but it may even promote membrane curvature. Although the mechanism(s) involved in this are still uncertain, the findings suggest roles for α-syn in the membrane bending necessary for vesicular exocytosis and recycling [95]. In cells, endogenous α-syn as well as the over-expression of this protein promotes the dilation of the fusion pore, and hence shifts exocytosis toward a higher prevalence of full vesicle fusion and a lower probability of kiss-and-run events [87,96]. This is dependent on the disordered region, as binding normal α-syn to the membrane makes dilation of the fusion pore energetically favorable, while a truncated version of α-syn, containing only the ordered regions (residues 1–100), failed to do so [97]. Furthermore, normal α-syn, but not the PD-associated variants where the vesicle-binding N-terminal is mutated, accelerates neuromodulator release by causing more complete fusion events. This may affect different neurotransmitters differently: Neuromodulators like the monoamines and neuropeptides dissociate slowly from vesicles and are therefore more likely to be affected by dilation of the fusion pore than the rapidly defusing amino acid transmitters. Indeed, α-syn over-expression or loss has been reported to affect the release of dopamine [83,98] but not the release of glutamate [99]. Further highlighting the complexity of the interaction between α-syn and the SV membrane, α-syn may be phosphorylated by G-protein coupled-receptor kinases (GRKs), especially GRK2 and GRK5. This reduces the phospholipid binding of α-syn and may contribute to the regulation of neurotransmitter release [100]. Immediately after synaptic activation, α-syn diffuses into the axon; this may result from flattening of the membrane and imply a role for α-syn before or in exocytosis, but presumably not in the subsequent endocytosis process.

In addition to the mainly presynaptic localization and function of α-syn, a recent article also found evidence for postsynaptic synuclein, where normal α-syn was found to be necessary for the release of the postsynaptic lipid transmitters endocannabinoids [101]. Mutant α-syn does not rescue endocannabinoid release in α-syn knockout mice [101]. The general dogma is that endocannabinoids are synthesized and release on demand [102,103], rather than via exocytotic release. Albarran and colleagues, however, found that the postsynaptic release of endocannabinoids was not only dependent on α-syn, but also postsynaptic SNAREs [101], indicating a vesicular release mechanism. Endocannabinoids are present across the brain, especially in the neocortex, hippocampus, basal ganglia, and cerebellum [104,105]. Endocannabinoids are released from the postsynaptic dendrites and work on presynaptic cannabinoid receptor 1 in a wide range of synapses, from glutamatergic and GABAergic, to dopaminergic and cholinergic [106,107,108]. The effect of α-syn on the release of endocannabinoids, however, has so far only been shown for glutamatergic and GABAergic synapses [101].

Collectively, these findings strongly indicate that α-syn plays a fundamental role in synaptic regulation. This regulation is essential for maintaining the delicate balance between excitation, inhibition, and neuromodulation in the brain. This involvement in synaptic homeostasis ensures that synaptic connections remain adaptable to changes, while preserving their core functionality. Remarkably, PD mutations affect this property without impairing the ability of α-syn to inhibit exocytosis, indicating a selective defect in normal function [96]. One hypothesis may be that fine-tuning normal α-syn function, in particular the binding of α-syn monomers to synaptic vesicles to regulate synaptic function, relies on a dynamic formation and degradation of oligomers. As monomers appear to be the main α-syn species to engage in the binding of curved membranes, reducing the number of available monomers through multimerization would presumably release the vesicles. In line with this, monomeric but not tetrameric α-syn has been shown to be able to bend membranes [109]. Further supporting this idea, the prevention of multimerization has been found to cause accumulation of α-syn monomers and increased membrane binding [33]. The authors of the latter study suggested that PD-related mutations of α-syn caused “constitutively active” monomers, which keep membrane-rich structures in aggregates, while multimers represented an “inactive storage form” of α-syn.

There is huge variability in α-syn content in different synapses, which may be indicative of variability of the normal function of α-syn across transmitter systems. Additionally, the endogenous amount of α-syn present in a synapse probably reflects—at least in part—whether that particular synapse is prone to be affected by α-syn aggregation in synucleinopathies. Supporting this correlation even further, studies have shown that neurons containing α-syn are more susceptible to reductions in spine density caused by exogenous α-syn proteofibrils. This effect was not observed in neurons without endogenous α-syn [110], suggesting that synapses that contain α-syn in the healthy brain are more prone to be affected during α-syn aggregation.

### 3.2. The Role of α-Syn in Dopaminergic Synapses

**Figure 3:**
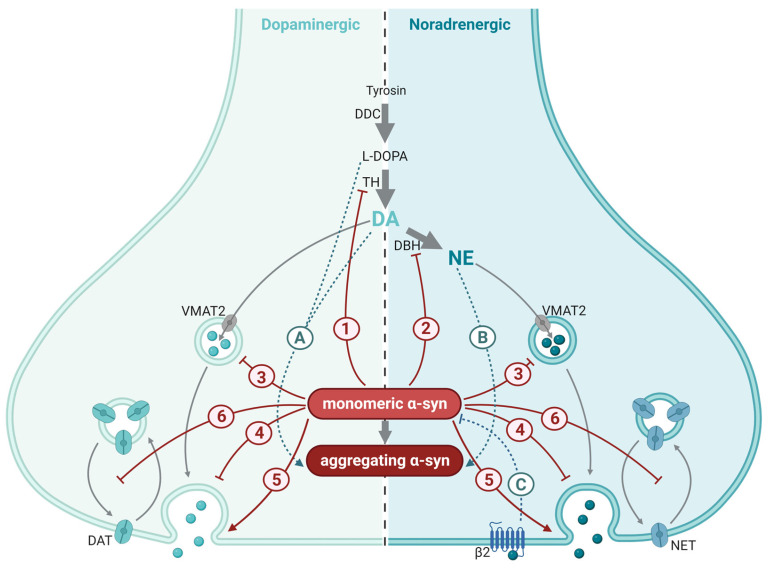
Synaptic mechanisms affected by α-synuclein (α-syn) in catecholaminergic synapses. Synthesis and synaptic mechanisms of dopamine (DA; (**left**)) and norepinephrine (NA; (**right**)) are indicated by gray arrows. Dopaminergic and norepinephrinergic neurotransmission is inhibited (red blunted arrows) or enhanced (red arrow) by α-syn through a myriad of mechanisms, most of which are shared between the two neurotransmitters: (1) inhibition of synthesis and activity of the rate-limiting enzyme of dopamine and norepinephrine synthesis, tyrosine hydroxylase (TH); (2) inhibition of synthesis of norepinephrine (NE) from dopamine (DA) via the enzyme dopamine β-hydoxylase (DBH); (3) reduced refilling rate of the readily releasable pool of synaptic vesicles; (4) reduced trafficking and re-clustering of SVs after neurotransmitter release; (5) enhanced dilation of the fusion pore; and (6) inhibition of the recycling of the dopamine transporter (DAT) and the norepinephrine transporter (NET) to the plasma membrane after transmission. The interaction between catecholamines and α-syn is bidirectional (turquoise dotted arrows): L-DOPA and DA (A), NE (B) along with their oxidized analogues stabilize α-syn protofibrils. (C) Activation of the β2 auto receptors on NE nerve terminals inhibits α-syn production.; DDC, dopa decarboxylase; VMAT2, vesicular monoamine transporter 2; β2, adrenergic receptor β2. This figure was created by the authors, using biorender.com.

Dopamine is synthesized from the essential amino acid tyrosine to L-3,4-dihydroxyphenylalanine (L-DOPA) by the enzyme tyrosine hydroxylase (TH) and further to dopamine by the enzyme DOPA decarboxylase (DDC; also known as amino acid decarboxylase, AADC). TH is the rate-limiting enzyme of this synthesis, and its activity is regulated at the transcriptional, translational, and post-translational levels, as well as through alternative RNA splicing and RNA stabilization. Short-term regulation of TH activity occurs mainly though phosphorylation, which occurs at Ser8, Ser19, Ser31, Ser40, and/or in the N-terminal regulatory region [111]. The regulation of TH activity, both short term and long term, may be impaired by α-syn. In cultured dopaminergic neurons, α-syn has been reported to inhibit the synthesis and the activation of TH (Figure 3), resulting in diminished production of transmitter DA. Furthermore, in silico data [112,113] suggest that α-syn docks at TH, causing a partial enfolding of the protein structure, which potentially reduces dopamine synthesis. Overexpression of normal α-syn has been reported to cause reduced activity of the TH promoter [114,115], with a subsequent reduction in TH both at the mRNA and protein levels [116]. The latter effect was reported for overexpression of the normal (A30P) variant of α-syn, but not for the (A53T) mutation known to cause familial PD. Confirming a link between α-syn levels and dopamine synthesis, stable silencing of α-syn in a mouse dopaminergic cell line (MN9D) was reported to enhance TH activation via phosphorylation, leading to increased levels of cytosolic L-DOPA and dopamine [117]. The interaction between α-syn pathology and catecholamines is bidirectional and has also been demonstrated at the stage of α-syn fibril formation: Screening 169 chemically different compounds, Conway and colleagues [118] identified 15 stabilizers of α-syn protofibrils, of which 14 turned out to be catecholamines. Among these were the neurotransmitters dopamine and noradrenalin, as well as their precursor L-DOPA. The same researchers found that dopamine—and presumably also the other catecholamines—stabilized protofibrils by producing covalent adducts with α-syn. Another study [119] confirmed the dopamine-induced inhibition of α-syn fibrils but suggested that oxidized dopamine by-products induce conformational changes in α-syn and that these changes make α-syn unable to assemble into mature fibrils. Interestingly, mutations in TH that resulted in a higher catalytic activity and higher intracellular dopamine levels enhanced the accumulation of potentially toxic α-syn oligomers in the cells. This was accompanied by progressive nigrostriatal degeneration and reduced locomotion. In contrast, mice expressing the same gain-of-function mutations in TH combined with unmutated α-syn did not show high levels of toxic α-syn oligomers and, hence, no PD-like symptoms [120]. The toxicity of α-syn oligomers is not understood in detail but α-syn pre-incubated with dopamine appears to inhibit SNARE complex formation, leading to reduced neurotransmission [110]; these species also seem to be resistant to chaperone-mediated autophagy [121]. The finding that oxidized dopamine analogues retain toxic α-syn protofibrils suggests a link between three key players in PD pathophysiology: dopamine, oxidative stress, and α-syn aggregation. Whether, or to what degree, this also occurs in vivo is not known. Another open question is whether the α-syn species that accumulate under reduced dopamine-induced stabilization of protofibrils (such as during PD) are able to drive neurodegeneration in the human brain.

Dopamine is transported into synaptic vesicles by VMAT2. Post mortem analysis of brain tissue revealed defects in VMAT2 in synaptic vesicles from PD patients, leading to a more-than-50% reduction in vesicular uptake of dopamine into VMAT2-positive vesicles in this patient group [78]. In line with this, findings from α-syn-deficient mice suggest that α-syn may negatively regulate dopamine release in part by modulating the refilling rate of the readily releasable pool of SVs [122].

Dopamine signaling is terminated by the reuptake of dopamine through high-affinity dopamine transporters (DATs) in the membrane of presynaptic terminals. Once dopamine is taken up into the presynaptic terminal through DAT, the DAT protein is internalized into the neuron through endocytosis. Efficient re-uptake depends on the constant recycling of the DAT protein back to the presynaptic membrane. This process is inhibited by α-syn through interactions with the microtubules [123,124].

### 3.3. The Role of α-Syn in Norepinephrine Synapses

Synthesis of norepinephrine starts with the conversion of tyrosine via L-DOPA to dopamine, as described above. The synthesis may be regulated by α-syn in norepinephrinergic neurons as well as in dopaminergic neurons. The enzyme dopamine β-hydroxylase (DBH), which is selectively expressed in norepinephrinergic neurons, further converts dopamine to norepinephrine. This enzyme is subjected to regulation at the transcriptional level, and cAMP response element (CRE) is a key positive regulator of the gene encoding DBH [125,126].

Both wild-type α-syn and α-syn with mutations known to be involved in human PD (A30P, A53T) have been suggested to interfere with the CRE-dependent regulation of DBH transcription in cultured norepinephrinergic cells [127] (Figure 3). While immobilization stress induced depression-like behavior and increased the number of DBH positive and TH positive cells in the locus coeruleus of wild-type mice, mice transgenically expressing A53T mutant α-syn failed to increase DBH or TH positive cells in response to this stressor. At the behavioral level, mice expressing A53T mutant α-syn scored higher in depression and anxiety tests [127]. Taken together, these findings indicate that inhibition of DBH by α-syn occurs in vitro as well as in vivo, at a level that is of functional and behavioral importance. Over-expression of α-syn in cells has been shown to disrupt recycling of the norepinephrine transporter (NET) to the plasma membrane, with a subsequent reduction in norepinephrine uptake into the cells [128]. Interestingly, the interaction between α-syn and norepinephrine signaling appear to be bidirectional: Screening 1126 FDA-approved drugs and other compounds, Mittal and co-workers identified four substances that lowered the production of SNCA mRNA and α-syn protein in cells. Three of these were agonists at β2-adrenorecetor, identifying this receptor as a regulator of α-syn production [129]. Supporting the correlation between β2 receptor activation and α-syn levels, an 11-year follow-up study of 4 million people showed that the intake of β2 receptor antagonist correlated with an increased risk of developing PD. Along the same lines, the anti-asthma drug salbutamol, which acts as a β2 receptor agonist and is able to cross the blood-brain barrier, was associated with a decreased risk of developing PD [129]. Although PD is regarded primarily as a dopamine deficiency syndrome confined to the nigrostriatal pathway, a deficiency in norepinephrine has been suggested to precede the deficiency in dopamine [9]. Supporting the hypothesis that altered norepinephrine metabolism plays a role in PD pathophysiology, DBH levels [130] and norepinephrine levels [131] have been found to be reduced in the CSF of PD patients.

### 3.4. The Role of α-Syn in Serotonergic Synapses

**Figure 4:**
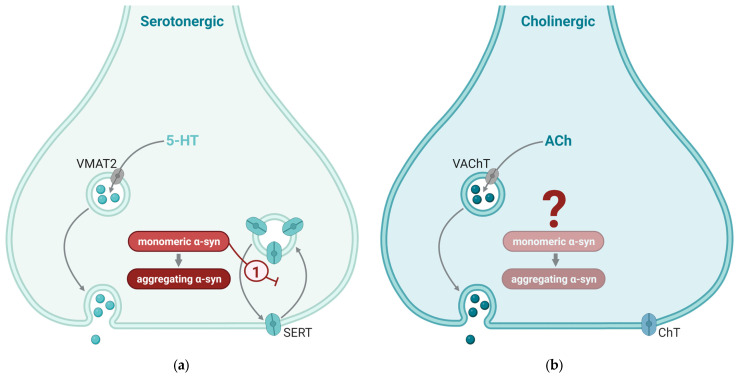
Synaptic mechanisms affected by α-synuclein (α-syn) in serotonergic and cholinergic synapses. Synaptic mechanisms of serotonin (5-hydroxytyrosine; 5-HT; (**a**)) and acetylcholine (ACh; (**b**)) are indicated by gray arrows. Mechanisms enhanced by α-syn are indicate by red arrows and mechanisms that are inhibited by α-syn are indicated by red blunted arrows. (**a**) Two key effects of α-syn in serotonergic neurotransmission are (1) enhanced dilation of the fusion pore and (2) inhibition of the recycling of serotonin transporter (SERT) to the plasma membrane after transmission. (**b**) Cholinergic nerve terminals generally have low α-syn levels; the effect of α-syn in these synapses is not well known. VMAT2, vesicular monoamine transporter 2; VAChT, vesicular acetylcholine transporter; SERT, serotonin transporter; ChT, choline transporter. This figure was created by the authors, using biorender.com.

Serotonergic neurons are localized to the raphe nuclei in the brainstem, from where they send ascending projections to most other brain regions and descending projections to the spinal cord. The raphe neurons of the human brain contain α-syn, which has been reported to affect these neurons (Figure 4). Serotonin released at the synapses is efficiently taken up into the presynaptic terminal through a serotonin transporter (SERT) in the plasma membrane. SERTs are expressed in synaptic areas of the widely projecting afferent fibers originating from the raphe nuclei and appear to be present in all regions of the human brain [132]. Dysregulation of SERT trafficking has been implicated in several neuropsychiatric disorders, including depression and anxiety disorders, and may partially be dependent on α-syn [84]. Structurally, the SERT protein is quite similar to DAT, and it is therefore not surprising that α-syn may interact with SERT in a similar way as described for DAT. Wersinger and co-workers [133] demonstrated that α-syn forms heteromeric complexes with SERT and, thus, prevents it from entering the plasma membrane. This effect is concertation dependent, resulting in a reduced uptake of serotonin in serotonergic neurons as intracellular α-syn levels increase. In an intact mouse brain, internasal administration of antisense oligonucleotides against α-syn, which selectively reduced α-syn expression in the monoamine nuclei of the brainstem, resulted in an increased effect of the SERT inhibitor citalopram [134]. In line with this, over-expression of wild-type human α-syn in serotonergic neurons produced the opposite effect (a lesser increase in extracellular serotonin in response to citalopram compared to the control mice). This was accompanied by progressive accumulation, phosphorylation, and aggregation of human α-syn protein in the serotonin system [135]. Further supporting this interaction between α-syn and SERT, transgenic mice where the human A53T mutant α-syn was expressed selectively in neurons showed reduced levels of serotonin in their raphe nuclei [136] along with a reduction in SERT-positive fibers to the dorsal hippocampus. The latter finding is in line with Lewy bodies and Lewy neurites being found post mortem in raphe neurons in PD patients [10].

### 3.5. The Role of α-Syn in Cholinergic Synapses

Acetylcholine is a neurotransmitter within both the central and the peripheral nervous systems. In the brain, acetylcholine facilitates distant signaling through projection neurons and local signaling via interneurons [137]. By affecting both metabotropic muscarinic and ionotropic nicotinic receptors, and undergoing both synaptic and extrasynaptic release [138], acetylcholine is capable of neuromodulation at multiple time scales.

Cholinergic activity in the brain arises from projection neurons extending to distant regions and widespread interneurons. Projection neurons, located in various brain nuclei such as the basal forebrain complex including the medial septum [139,140], the medial habenula [141], and laterodorsal tegmental area and PPN. Cholinergic interneurons exist both in the basal ganglia and in the rodent [142] and human neocortex [143,144].

Staining for α-syn in many cholinergic areas, such as the medial and lateral septum within the basal forebrain [145] is generally weak, suggesting a less prominent role of α-syn in cholinergic synapses as a whole (Figure 4). There is a notable absence of studies examining the co-localization of α-syn and acetylcholine in healthy brains, but specifically within the PPN—a nucleus deeply intertwined with both the clinical manifestations and neuropathological characteristics of PD [146]. The PPN encompasses a diverse neuronal population consisting of approximately 40% glutamatergic, 35% GABAergic, and 25% cholinergic neurons [147]. Studies suggest that synapses harboring α-syn in healthy brains are prone to α-syn aggregation in synucleinopathies [110]. Building upon these insights, we anticipate a comparable scenario in cholinergic synapses within the PPN. This is reinforced by observations showing that, despite their lower prevalence within the PPN, cholinergic neurons exhibited the predominant phosphorylated α-syn after exposure to preformed α-syn fibrils [147]. Pathological α-syn specifically accumulates within cholinergic neurons in the PPN, without being observed in neighboring noncholinergic neurons. The emergence of phosphorylated α-syn pathology coincided with a notable reduction in choline acetyltransferase immunoreactivity, implying a potential decline in cholinergic neurons, whereas markers for other neuronal types remained unchanged [147].

### 3.6. The Role of α-Syn in Glutamatergic Synapses

While α-syn is present in glutamatergic synapses, indications point to varying roles of α-syn between the different glutamatergic synapses across the brain. Exogenous α-syn is shown to affect the mobilization of distinct SV pools and organization of active zone components in glutamatergic synapses though cholesterol binding [148], further leading to increased tonic and decreased depolarization-evoked SV recycling and glutamate release [148]. There is also a distinction in the amount of α-syn between the vesicular glutamate transporter 1 (VGLUT1) positive terminals and VGLUT2 positive terminals. In, e.g., mouse amygdala, where most of the glutamatergic neurons are VGLUT1 positive, there is much more α-syn than in the VGLUT2 positive glutamatergic neurons in the mouse thalamus [149]. The difference in α-syn levels between the VGLUT1 positive terminals in amygdala and the VGLUT2 positive terminals in thalamus is further supported by reduced activity in cortico-amygdalal synapses in α-syn knockout mice [149]. Additionally, when α-syn aggregates, leaving a reduced amount of soluble α-syn, the transmission in VGLUT1 synapses in amygdala, but not the VGLUT2 synapses in thalamus, is reduced [149]. Although the general tendency is that more α-syn is present in VGLUT1 terminalis compared to VGLUT2 terminals, in some regions, like substantia nigra pars compacta, there are both α-syn positive and negative VGLUT1 synapses [150].

Also, aggregating α-syn affects pathways or proteins that are specific for glutamatergic synapses. In transgenic mice expressing human α-syn, α-syn aggregation leads to an increased expression of the metabotropic glutamate receptor 5 (mGluR5) postsynaptically and an increased ratio of the N-methyl-d-aspartate (NMDA) receptor subunits GluN1/GluN2A [151]; both of these changes normalized when treated with a protein aggregation inhibitor [151]. Aggregating α-syn could also induce calcium-dependent glutamate release from astrocytes, and glutamate-mediated activation of the glutamate receptor NMDAR extrasynaptically [152]. In patch-clamp experiments, oligomerized α-syn is shown to directly activate NMDARs [152]. The activation of extrasynaptic NMDARs by α-syn further mediated synapse loss in both organotypic cultures and human induced pluripotent stem cell-derived cerebrocortical neurons; this was reversed through the addition of an NMDAR antagonist [152]. In a healthy brain, α-syn does not seem to be obligatory for long-term potentiation (LTP), the neurophysiological equivalent of memory and learning, as robust NMDAR-dependent LTP was equally present in wild-type and α-syn-deficient mice [68]. NMDAR activation, triggering an enhanced contribution of calcium-permeable AMPA receptors, however, was enhanced through exposure to α-syn oligomers, but not fibrils [153].

**Figure 5:**
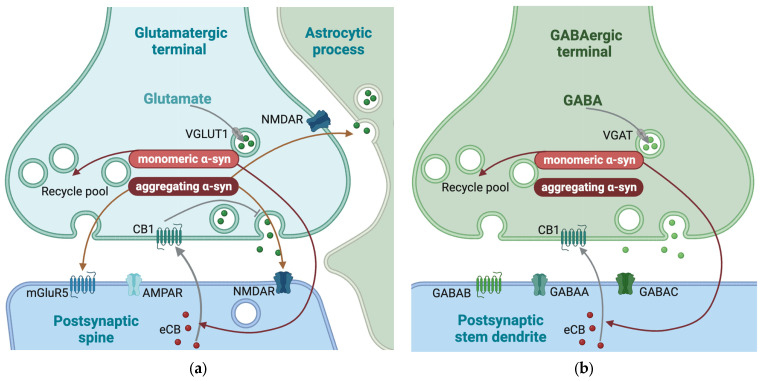
Synaptic mechanisms affected by α-synuclein (α-syn) in glutamatergic and GABAergic synapses. Mechanisms enhanced by α-syn are indicate by red arrows. (**a**) In glutamatergic synapses, monomeric α-syn is important for the maintenance of the size of the recycling pool of synaptic vesicles. Additionally, aggregating α-syn increases the ratio of the *N*-methyl-d-aspartate (NMDA) receptor subunits GluN1/GluN2A and the expression of metabotropic glutamate receptor 5 (mGluR5) and contributes to astrocytic glutamate release. (**b**) The main effects of α-syn in GABAergic neurotransmission are on the maintenance of the size of the recycling pool of synaptic vesicles. Monomeric α-syn is also shown to be necessary for postsynaptic release of endocannabinoids (eCB) in both glutamatergic and GABAergic synapses. The eCB activate (gray arrow) presynaptic cannabinoid receptor 1 (CB1) which, in glutamatergic synapses leads to inhibition (grey blunted arrow) of exocytosis. VGLUT1, vesicular glutamate transporter; VGAT, vesicular GABA transporter; CB1, cannabinoid receptor 1; GABAA, GABA type A receptor; GABAB, GABA type B receptor; GABAC, GABA type C receptor. This figure was created by the authors, using biorender.com.

In addition to the direct effect on glutamatergic vesicles and metabotropic and ionotropic glutamate receptors, the postsynaptic release of endocannabinoids, inhibiting presynaptic glutamate release, are shown to be α-syn-dependent [101] (Figure 5).

### 3.7. The Role of α-Syn in GABAergic Synapses

The presence of α-syn in GABAergic synapses differs among brain regions. While α-syn is mainly localized in inhibitory terminals in *substantia nigra pars reticulata*, it is mainly located to excitatory terminals in the cerebral cortex [150]. In addition to *substantia nigra pars reticulata*, α-syn has also been reported in GABAergic terminals in the external and internal globus pallidus and the external plexiform layer of the olfactory bulb. However, α-syn has not been reported in GABAergic terminals in the cerebral cortex or hippocampus [154], subthalamic nucleus, or thalamus [150]. Supporting the notion that synapses without α-syn in healthy brains are better protected when α-syn starts to aggregate, most of the GABAergic synapses in the hippocampus are reported to be free of Lewy body aggregates in conditions where Lewy bodies were found in other types of neurons [155]. However, it was possible to induce Lewy body formation by overexpressing human α-syn in the same GABAergic neurons, indicating that the low amount of Lewy bodies in GABAergic terminals may be linked to the low amount of naturally occurring α-syn in the inhibitory neurons [150]. As described for glutamate above, the postsynaptic release of endocannabinoids, which inhibit presynaptic GABA release, has been shown to be α-syn-dependent [101] (Figure 5).

As reported for most other synapse types, α-syn contribute to the maintenance of the size of the recycling pool of SVs in both glutamatergic and GABAergic neurons [156]. Both the recycling pool and total SV pool are reported to be more variable in glutamatergic synapses compared to GABAergic synapses [157]. This heterogeneity of the recycling pool sizes among glutamatergic synapses may create a dynamic range for synaptic strength, a variability not seen among GABAergic synapses [157,158]. Based on this, it has been hypothesized that the role of α-syn in glutamatergic synapses is mainly as a modulator of the recycling pool.

### 3.8. Converging and Diverging Mechanisms

**Figure 6:**
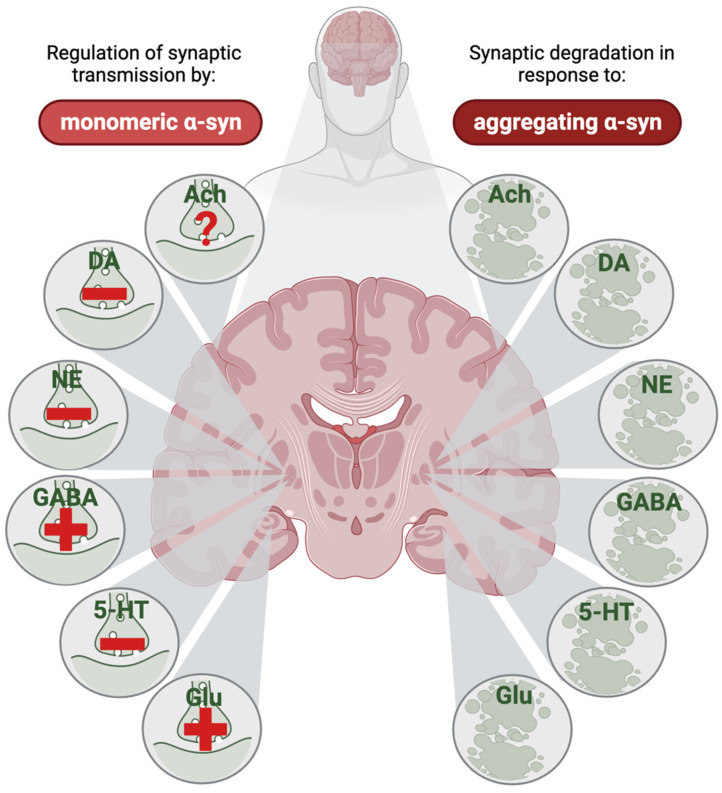
The net effect of monomeric α-synuclein (α-syn) on neurotransmission and of aggregating α-syn on synaptic degeneration in different neurotransmitter systems. In a healthy brain, α-syn affects neurotransmission through different mechanisms, and the net effect of this differs between different transmitter systems; in some synapses, α-syn triggers enhanced neurotransmission (indicated by a plus-sign in the left part of the figure), whereas in other synapses, α-syn reduces neurotransmission (indicated by a minus sign in the left part of the figure). Evidence from animal and human research are combined in this illustration, assuming a similar effect of monomeric α-syn on transmitter dynamics in the synaptic terminals in healthy brains across species. The direct effect of monomeric α-syn on cholinergic transmission is, to the best of our knowledge, yet undetermined (indicated by a question mark in the left part of the figure). When α-syn starts to aggregate, degeneration of all these types of synapses has been reported (right part of the figure), although some of the evidence are indirect through reductions in transmitter systems seen in Parkinson’s disease. For many of the transmitter systems, the cell body-containing nuclei in the brain stem have been in focus; but in this illustration, the respective synapses are marked at one of several places where these projection form synapses, like in the putamen for dopaminergic and norepinephrinergic neurons, the external globus pallidus for GABAergic and serotonergic neurons, the cerebral cortex for cholinergic neurons, and the hippocampus for glutamatergic synapses. Ach, acetylcholine; DA, dopamine; NE, norepinephrine; GABA, gamma amino butyric acid; 5-HT, serotine; Glu, glutamate. This figure was created by the authors, using biorender.com.

Among the synaptic mechanisms that are affected by α-syn, some are general and shared across all synapses where the protein is expressed. Others are specific for one or more neurotransmitter systems or a subset of synapses. In addition, expression levels of α-syn differ between synapses, and this seems to determinate to what degree synaptic functions are affected by α-syn-dependent mechanisms. Consequently, a large heterogeneity in the net effects of α-syn in different synapse types is observed (Figure 6). Among the converging mechanisms is the ability to interact with a number of priming proteins to enhance the formation of the SNARE complex [74,75,84,90,91,159,160]. This would, in theory, enhance exocytosis in all nerve terminals, but is likely proportional to the amount of α-syn present. The same holds true for the propensity to bind curved membranes of the SVs and cause dilation of the fusion pore. Through this mechanism, α-syn shifts exocytosis towards more events of complete fusion instead of the partial fusion involved in the “kiss-and-run” release [87,95,96]. Despite being a common mechanism, it may affect different neurotransmitters in different ways. The small amino acid transmitters can easily escape through the narrow aqueous fusion pore created during a “kiss-and-run” event, and are therefore largely unaffected by this effect of α-syn [99]. The monoamines, on the other hand, are larger in size and will diffuse more slowly through a narrow fusion pore [83,98]. By enhancing the probability for complete fusion events, α-syn therefore causes a greater effect on the release of these transmitters. Modulation of different pools of vesicles is also likely a shared mechanism, but the literature is not consistent regarding the effects of α-syn on SV cycling between these pools [68,69,81,82,83]. Furthermore, the size of each pool likely differs between synapses, causing for diverging effects across transmitter systems and subsets of synapses.

Counteracting the enhanced release induced by the general mechanisms presented above, transmission of the catecholamines is inhibited at the level of synthesis. The protein levels and activity of several enzymes involved in the production of dopamine and norepinephrine are inhibited by α-syn [111,112,113,114,115,116,117,125,126,127].

A converging mechanism for α-syn in monoaminergic synapses is the regulation of reuptake. Reuptake of monoamines is handled by the plasma membrane transporters DAT, NET, or SERT. Internalization of these transporters via endocytosis and their recycling back to the presynaptic membrane regulate reuptake capacity. Recycling of all three transporter proteins is inhibited by α-syn [123,124]; this contributes to a reduced reuptake and an augmented effect of the respective neurotransmitters. Extracting the net effect of α-syn on neurotransmission is not an easy task. For the catecholamines, it appears that the inhibition of synthesis outcompetes the mechanisms that lead to enhanced exocytosis, so that the overall effect is a reduction in dopaminergic and norepinephrinergic submission. Supporting the conclusion that dopamine and norepinephrine transmission is reduced, the levels of these transmitters are reduced in the CSF of PD patients [131]. This conclusion is, however, precluded by that fact that catecholaminergic neurons selectively die in patients suffering from this disease. For serotonin, the inhibition of SERT recycling [84,133,134,136], the general regulation of vesicular pools, and the dilation of the fusion pore all pull towards enhanced neurotransmission. The effects of α-syn in the cholinergic system are hard to interpret, as α-syn levels appear to be low [145,146], and few studies have investigated whether cholinergic neurotransmission is affected by α-syn. As exocytotic release of the amino acid transmitters, glutamate, and GABA appear to be largely unaffected by α-syn-induced dilation of the fusion pore [99], the effect of α-syn in these nerve terminals mainly reflects the redistribution of synaptic vesicles [156,157]. In itself, this does not affect normal neurotransmission, but in glutamatergic synapses of the hippocampus, prolonged low-frequency stimulation sufficient to deplete the reserve pool is higher in wild-type mice than in α-syn-deficient mice [69,81,82]. A profound heterogeneity of endogenous α-syn levels as well as of the recycling pool sizes among glutamatergic synapses [157,158] presumably underlie a differential effect of α-syn in these synapses. Adding to the complexity, α-syn at glutamatergic and GABAergic synapses is necessary for a postsynaptic release of the retrograde endocannabinoid transmitters [101], which bind to presynaptic receptors to inhibit further release of glutamate or GABA. Nevertheless, the net effect of α-syn in glutamatergic and GABAergic synapses appears to be an enhanced release.

Regarding the toxic effects of α-syn in diseases involving synucleinopathies, it appears that aggregates of α-syn are related to the amount of endogenously expressed α-syn in each synapse type. Hence, the synapses who express high amounts of α-syn are likely affected at some stage of disease, while synapses with low expression levels appear to be protected (Figure 6). Nevertheless, the catecholamines appear to be in a special situation, as there is strong evidence suggesting that these transmitters reinforce α-syn toxicity.

## 4. Conclusions

The abundant presence of α-syn in synapses and its effect on general synaptic functions as well as on individual neurotransmitter systems of a healthy brain underscores its significance beyond the context of neurodegenerative diseases. While the precise roles of α-syn are still the subject of ongoing investigations, it is evident that this protein is not merely a bystander but an active participant in normal brain function. Fully understanding the physiological functions of α-syn in a healthy brain could potentially lead to innovative therapeutic strategies for neurodegenerative diseases which are characterized by the misfolding and aggregation of α-syn. Different synapses in the brain differ in their content of α-syn, which correlates with the susceptibility of these synapses to α-syn aggregation in patients who develop synucleinopathy.

## Data Availability

No new data were created or analyzed in this study. Data sharing is not applicable to this article.
